# Treatment of clinical T4 stage superior sulcus non-small cell lung cancer: a propensity-matched analysis of the surveillance, epidemiology, and end results database

**DOI:** 10.1042/BSR20181545

**Published:** 2019-02-01

**Authors:** Junmiao Wen, Di Liu, Donglai Chen, Jiayan Chen, Xinyan Xu, Chang Chen, Fuquan Zhang, Shanzhou Duan, Rongying Zhu, Min Fan, Yongbing Chen

**Affiliations:** 1 Department of Radiation Oncology, Fudan University Shanghai Cancer Center, Shanghai, China; 2 Department of Oncology, Shanghai Medical College, Fudan University, Shanghai, China; 3 Department of Thoracic Surgery, Shanghai Pulmonary Hospital, Tongji University, School of Medicine, Shanghai, China; 4 Department of Cardiothoracic Surgery, the Second Affiliated Hospital of Soochow, University, Medical College of Soochow University, Suzhou, China

**Keywords:** radiotherapy, SS-NSCLC, stage IV, surgery

## Abstract

**Purpose/Objective(s):** Treatments for superior sulcus non-small cell lung cancer (SS-NSCLC) have evolved, but adequate treatments of T4 disease have not been found. The aim of our study was to evaluate the prognostic factors and optimal treatment strategy for patients with T4 SS-NSCLC. **Materials/Methods:** We utilized the Surveillance, Epidemiology, and End Results (SEER) database (1973–2015) to identify patients diagnosed with T4 stage SS-NSCLC (according to the 7th edition American Joint Committee on Cancer (AJCC) staging system) from 2004 to 2015; those with M1 disease were excluded. Propensity score matching (PSM) with Kaplan–Meier and Cox proportional hazards’ models was performed to estimate prognosis. **Results:** A total of 384 patients were included. The majority was male (59.4%) at stage IIIB (56.6%), with N2 accounting for 45.3%. A total of 47 patients underwent cancer-directed surgery, while radiotherapy alone was received by 60.2% of patients. Median overall survival (OS) and lung cancer-specific survival (LCSS) were 12 and 17 months, respectively, and the 5-year OS and LCSS rates were 15.8 and 25.4%, respectively. In the matched population, the median survival outcomes were better following surgery (OS: 25 compared with 9.0 months, *P*<0.001; LCSS: not available (NA) compared with 11.0 months, *P*<0.001). Multivariate Cox analysis showed that ages ≥ 66 years (hazard ratio (HR) = 1.639, *P*=0.001), unmarried status (HR = 1.356, *P*=0.034), and tumor size ≥ 6.0 cm (HR = 1.694, *P*<0.001) were associated with inferior OS. Cancer-directed surgery (HR = 0.537, *P*=0.009) and radiotherapy (HR = 0.644, *P*=0.006) were independent prognostic factors for patients with T4 SS-NSCLC. Conversely, in the subgroup analysis, favorable impacts of radiotherapy were observed for nonsurgical patients (OS: HR = 0.58, *P*<0.001; LCSS: HR = 0.55, *P*<0.001). **Conclusion:** Our study showed that T4 stage SS-NSCLC patients had a poor prognosis. Surgical resection remains the best option for those with resectable disease. For nonsurgical T4 SS-NSCLC patients, radiotherapy should be actively considered.

## Introduction

Superior sulcus non-small cell lung cancer (SS-NSCLC), a unique subgroup of locally advanced NSCLC that originates in the apex of the lung, poses a great challenge for clinicians [1,2]. The involvement of adjacent critical structures, including the spines, brachial plexus, and subclavian vessels, makes a complete surgical resection with negative margin extremely difficult [2]. The management of SS-NSCLC has evolved over time, with the standard multimodality approach resulting in the best loco-regional control and long-term survival. Induction chemoradiotherapy (CRT) followed by surgery has been demonstrated to be safe and effective for marginally resectable SS-NSCLC patients by the phase II trials Southwest Oncology Group (SWOG) 9416 [3] and Japan Clinical Oncology Group (JCOG) 9806 [4]. In addition, a prospective phase II study has reported favorable results for SS-NSCLC patients receiving surgery followed by CRT [5]. Thus, the current Nation Comprehensive Cancer Network (NCCN) guidelines recommend multimodality treatment using concurrent CRT followed by surgery for SS-NSCLC patients at stage T3-4N0-1 [6].

Although the outcomes for SS-NSCLC have improved, several questions remain unsolved. One question concerns management of patients with mediastinal node involvement. In the studies cited above, the majority of enrolled patients had good performance status (PS 0-1) and staged as T3-4N0-1. In the recent phase II trial that added consolidation chemotherapy after induction of CRT followed by surgery, patients with N2 disease were excluded [7]. In addition, T4 disease accounted for 20–30% of SS-NSCLC patients, with frequent occurrence of distant metastasis and dismal prognosis [4,8–10]. Also, in the SWOG 9416 and JCOG 9806 trials, complete resection could be achieved in less than half of the patients with T4 disease, and the surgical treatment requires the participation of a multidisciplinary surgical team, which varies widely amongst different institutions [2,10,11]. Furthermore, in the previously published studies, T4 disease was always a small proportion of all disease, and only limited data are available in the literature on the resection rate as well as the optimal treatment of T4 disease with regard to the N stage [7,12].

In this context, we performed the present database analysis to investigate the roles of surgery and/or radiotherapy in different sequences and identified the potential prognostic factors for patients with T4 SS-NSCLC using the Surveillance, Epidemiology, and End Results (SEER) database.

## Materials and methods

### Study population

We conducted this retrospective study using data retrieved from the SEER database supported by the National Cancer Institute, which provides information about cancer patients in 18 registries of the U.S. and covers approximately 28% of the U.S. population [13]. Tumors of the lung were identified with the primary site code C34.0-34.9. The eligibility criteria in the present study included: (i) age older than 18 years and lung was the first primary site; (ii) diagnosed between 2004 and 2015; (iii) survival time ≥ 1 month; (iv) histologically confirmed NSCLC ( International Classification of Diseases for Oncology 3rd Edition (ICD-O-3) codes: 8050–8052, 8070–8078, 8140–8147, 8250–8255, 8260, 8310, 8430, 8480, 8481, 8571–8575, and 8012–8013); (v) CS tumor extension code 610 (superior sulcus tumor) [14]. Patients with M1 stage were excluded (according to the 7^th^ edition of the American Joint Commission on Cancer Staging Manual) [15]. Demographic features and clinicopathological characteristics of these patients were collected (Table 1). The study was approved by the institutional review board and independent ethics committee of Fudan University Shanghai Cancer Center (Shanghai, China).

**Table 1:** Basic characteristics of T4 SS-NSCLC patients

Characteristics	SST NSCLC	
	(*n*=384)	%
**Sex**		
Male	228	59.4
Female	156	40.6
**Age (years)**		
Median (range)	66	29–95
Mean (S.D.)	66.35	±11.71
**Ethnicity**		
Caucasian	325	84.6
African	39	10.2
Other	20	5.2
**Marital status**		
Married	187	50.3
Unmarried	185	49.7
Unknown	12	
**Region (CHSDA)**		
East	193	50.3
Pacific Coast	132	34.4
Northern Plains	44	11.5
Southwest	15	3.9
**Grade**		
Well/Moderate	63	30.7
Poor/Undifferentiated	142	69.3
Unknown	179	
**Location**		
Left	201	52.3
Right	178	46.4
Bilateral	5	1.3
**Histological type**		
SQ	136	35.4
AD	96	25.0
Others*	152	39.6
**AJCC stage**		
IIIA	160	43.4
IIIB	209	56.6
Unstaged	15	
**N stage**		
N0	130	35.2
N1	30	8.1
N2	167	45.3
N3	42	11.4
NX	15	
**Tumor size (cm)**		
Median (Range)	6.0	3-18
Mean (SD)	6.1	±2.8
**Surgery**		
Yes	47	12.2
No	337	87.8
**Surgery type**		
Partial wedge resection	16	34.0
Lobectomy	25	53.2
Pneumonectomy	6	12.8
**Radiotherapy**		
Yes	257	66.9
No	127	33.1
**Treatment type**		
None	106	27.6
Surgery only	21	5.5
RT only	231	60.2
NRT	15	3.9
PORT	11	2.9

*Others in the histological type included: epithelial neoplasms, cystic, mucinous and serous neoplasms, complex epithelial neoplasms and complex mixed and stromal neoplasms. Abbreviations: AD, adenoma and adenocarcinoma; AJCC, American Joint Committee on Cancer; CHSDA, Contract Health Service Delivery Area; NRT, neoadjuvant radiotherapy; PORT, postoperative radiotherapy; RT, radiotherapy; SQ, squamous cell neoplasm.

### Subset definitions

In the present study, included patients were categorized by sex, age, ethnicity, marital status, differentiation grade, histological type, tumor location, stage and tumor size. Histological subgroups were defined as squamous cell carcinoma, adenocarcinoma, and other types according to the ICD-O-3 [16]. Surgical types were grouped into partial wedge resection, lobectomy, and pneumonectomy. In addition, based on the different treatment modalities, we classified the type of treatment into five categories: surgery only, radiotherapy only, postoperative radiotherapy (PORT), neoadjuvant radiotherapy (NRT), and no treatment performed.

### Statistical analysis

To adjust for the difference between patients undergoing surgery and those who did not, similar to a previous study [17], a one-to-one propensity-matched analysis was performed. The propensity score matching (PSM) model was based on the demographic features mentioned above. Continuous variables were compared using Student’s *t*test or the nonparametric Mann–Whitney U test as indicated. Frequencies and proportions were calculated for categorical variables and chi-square tests or Fisher’s exact tests were adopted to compare between groups. Kaplan–Meier survival curves were assessed by log-rank test. One end point of the present study was overall survival (OS), which was defined as the interval between the diagnosis of cancer and death. Another end point, lung cancer-specific survival (LCSS) was measured from diagnosis until death from SS-NSCLC. Univariate analyses were conducted on all variables included in the study. In the univariate analysis, variables with *P*<0.2 were entered into the multivariate analyses. We employed the multivariate Cox regression model for OS and LCSS to estimate the prognostic effect of the covariate. Two-tailed *P*-values <0.05 were considered statistically significant. All statistical analysis was performed with IBM SPSS Statistics 22.0 (IBM, Armonk, NY, U.S.A.) and GraphPad Prism 7.0 (GraphPad Software, San Diego, CA, U.S.A.).

## Results

### Clinicopathological characteristics

A total of 384 patients during 2004–2015 were enrolled. The characteristics and demographics of the cohort are outlined in Table 1. Overall, most patients were male (59.4%), Caucasian (84.6%), married (50.3%), and had stage IIIB disease (56.6%) according to the American Joint Committee on Cancer (AJCC) 7th staging system. The median patient age was 66 years (range: 29–95 years). Primary tumors were usually large (40.6%) and arose in the left lung (52.3%), with a median size of 6 cm (range: 3–18 cm). As for N stage, most patients were diagnosed with N2 stage (167, 45.3%), followed by N0 (130, 35.2%), N3 (42, 11.4%), and N1 (30, 8.1%). Poor or undifferentiated pathological grade (69.3%) was diagnosed in majority of patients. Squamous cell carcinoma (35.4%) was the most common histologic subtype followed by adenocarcinoma (25%) in the entire population.

In terms of treatment, only 47 patients (12.2%) received surgery or NRT/PORT (N0 = 26, N1 = 5, N2 = 15, N3 = 1), while a large percentage of patients (231, 60.2%) underwent radiotherapy only. The resection rate was 20, 16.7, 9, and 2% in N0, N1, N2, and N3 patients, respectively. The data seemingly indicated that patients with Caucasian ethnicity, squamous cell histology type, IIIA stage, without lymph node metastases, and smaller tumor size were more likely to undergo surgical resection. Of the 47 surgical patients, 25 (53.2%) received lobectomy, 16 (34.0%) received partial wedge resection, and 6 (12.8%) were pneumonectomized. Meanwhile, radiotherapy was delivered to the majority of patients who did not undergo surgery (231 patients, 68.5%, Supplementary Table S1). As shown in Supplementary Table S1, a 1:1 PSM was performed between patients with or without surgery. After PSM, neither ethnicity, histological type, AJCC stage, N stage, tumor size nor radiotherapy differed significantly between the two subgroups.

### Survival analysis

**Figure 1:**
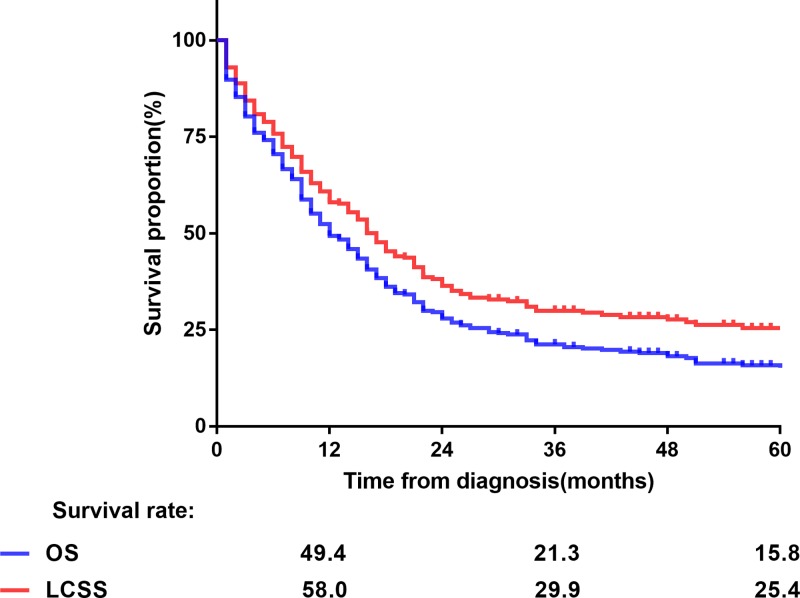
OS and LCSS for T4 SS-NSCLC patients

**Figure 2:**
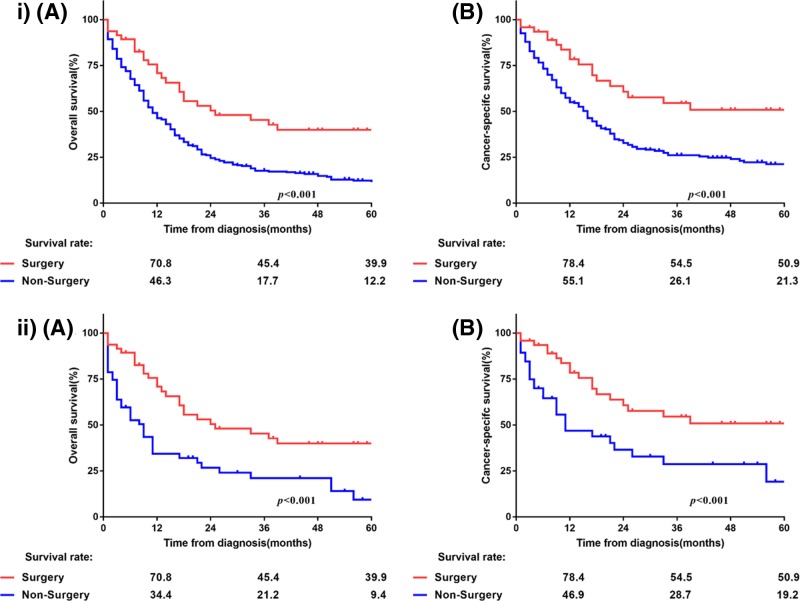
OS (**A**) and LCSS (**B**) according to the administration of surgery: (i) in the entire cohort, (ii) in the matched population

**Figure 3:**
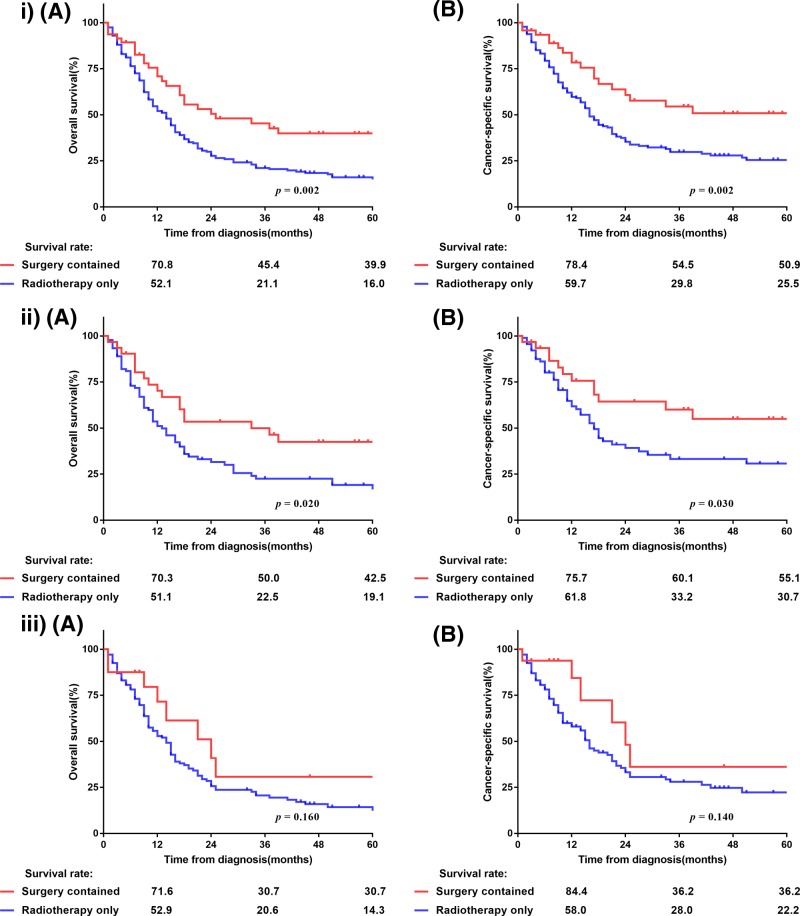
OS (**A**) and LCSS (**B**) of T4 SS-NSCLC patients received surgery contained treatment (including surgery only, PORT and NRT) or radiotherapy alone: (i) in the entire cohort, (ii) N0-1 subgroup, (iii) N2-3 subgroup

**Figure 4:**
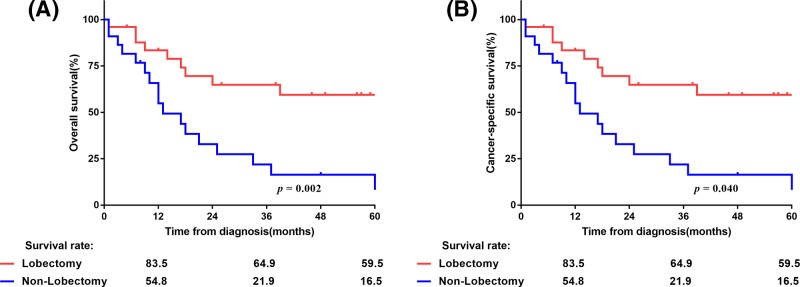
OS (**A**) and LCSS (**B**) in patients underwent surgery according to the administration of lobectomy

With a median follow-up of 10 months (range: 1–126 months), the median OS and LCSS were 12 and 17 months, respectively, with 1-, 3- and 5-year OS rates of 49.4, 21.3, and 15.8%, respectively, and 1-, 3- and 5-year LCSS rates of 58, 29.9, and 25.4%, respectively (Figure 1). The Kaplan–Meier survival curves of matched populations based on the administration of surgery are displayed in Figure 2. In the propensity-matched cohort, those who underwent surgery had significantly better survival compared with those without surgery (OS: 25 compared with 9 months, *P*<0.001; LCSS: not available (NA) compared with 11 months, *P*<0.001). Similarly, patients who underwent radiotherapy only had worse OS and LCSS than surgical patients in the entire population (OS: 14 compared with 25 months, *P*=0.002; LCSS: 16 months compared with NA, *P*=0.002), as well as in the N0-1 subgroup (OS: 13 compared with 33 months, *P*=0.02; LCSS: 17 months compared with NA, *P*=0.03; Figure 3). However, the survival difference was not significant in patients with N2-3 disease (OS: 24 compared with 14 months, *P*=0.16; LCSS: 24 compared with 16 months, *P*=0.14; Figure 3). Amongst 47 patients who received surgery, the median OS and LCSS of patients who received lobectomy was 71.4 and 75.1 months, respectively, which was significantly superior to the OS and LCSS in those who had undergone partial wedge resection or pneumonectomy (27.8 and 47.2 months, respectively; *P*=0.002 and *P*=0.04; Figure 4). Nevertheless, neither adjuvant nor NRT was an independent prognostic factor (*P*>0.05, Supplementary Figure S1). Because there were overlaps in the survival curves amongst the three groups, landmark analyses of OS and LCSS at 12 and 24 months were performed to minimize lead time bias [18], and the results confirmed the landmark analyses (Supplementary Table S2).

### Prognostic factors

Table 2 lists the results of univariate and multivariate analyses for OS and LCSS in the entire cohort. In the multivariate Cox analysis, age ≥ 66 years (hazard ratio (HR) = 1.6, 95% confidence interval (CI): 1.2–2.2, *P*=0.001), unmarried status (HR = 1.4, 95% CI: 1.0–1.8, *P*=0.034), and tumor size ≥ 6.0 cm (HR = 1.7, 95% CI: 1.3–2.3, *P*<0.001) were associated with inferior OS. Cancer-directed surgery (HR = 0.5, 95% CI: 0.3–0.9, *P*=0.009) and radiotherapy (HR = 0.6, 95% CI: 0.5–0.9, *P*=0.006) were independent protective factors. Multivariate Cox analysis for LCSS showed that patients with tumor size ≥ 6.0 cm (HR = 1.8, 95% CI: 1.3–2.5, *P*=0.001) had significantly worse prognosis. Significantly superior LCSS was seen in patients with surgical resection (HR = 0.5, 95% CI: 0.3–0.9, *P*=0.026) and radiotherapy (HR = 0.6, 95% CI: 0.4–0.9, *P*=0.006).

**Table 2:** Results of univariate and multivariate Cox regression of prognostic factors for OS and LCSS in T4 SS-NSCLC patients (384 patients)

Characteristics	OS								LCSS							
	Univariate analysis	95% CI lower	95% CI upper	*P*	Multivariate analysis	95% CI lower	95% CI upper	*P*	Univariate analysis	95% CI lower	95% CI upper	*P*	Multivariate analysis	95% CI lower	95% CI upper	*P*
**Sex**																
Male	Reference				-	-	-	-	Reference				-	-	-	-
Female	0.954	0.752	1.209	0.695	-	-	-	-	0.934	0.711	1.226	0.621	-	-	-	-
**Age (years)**																
<66	Reference				Reference				Reference				-	-	-	-
≥66	1.364	1.079	1.725	0.009	1.639	1.214	2.213	0.001	1.092	0.837	1.425	0.515	-	-	-	-
**Ethnicity**																
Caucasian	Reference				-	-	-	-	Reference							
African	1.176	0.813	1.701	0.389	-	-	-	-	1.209	0.795	1.837	0.375	1.159	0.705	1.906	0.560
Other	1.362	0.778	2.385	0.279	-	-	-	-	1.644	0.915	2.954	0.097	1.482	0.768	2.861	0.241
**Marital status**																
Married	Reference				Reference				Reference							
Unmarried	1.291	1.021	1.632	0.033	1.356	1.023	1.798	0.034	1.263	0.966	1.65	0.087	1.219	0.883	1.683	0.230
**CHSDA**																
East	Reference				-	-	-	-	Reference				-	-	-	-
Pacific Coast	1.058	0.815	1.374	0.671	-	-	-	-	1.041	0.773	1.401	0.793	-	-	-	-
Northern Plains	1.147	0.794	1.657	0.464	-	-	-	-	1.056	0.686	1.626	0.804	-	-	-	-
Southwest	0.89	0.482	1.642	0.709	-	-	-	-	0.842	0.411	1.725	0.639	-	-	-	-
**Grade**																
Well/Moderate	Reference				-	-	-	-	Reference				-	-	-	-
Poor/Undifferentiated	0.935	0.657	1.332	0.710	-	-	-	-	0.921	0.619	1.371	0.685	-	-	-	-
**Location**																
Left	Reference				-	-	-	-	Reference				-	-	-	-
Right	0.957	0.757	1.210	0.713	-	-	-	-	1.094	0.838	1.429	0.509	-	-	-	-
**Histological type**																
Others	Reference				-	-	-	-	Reference				Reference			
SQ	0.852	0.653	1.112	0.240	-	-	-	-	0.802	0.593	1.084	0.151	0.981	0.681	1.413	0.919
AD	0.781	0.577	1.058	0.111	-	-	-	-	0.697	0.491	0.99	0.044	0.869	0.567	1.333	0.520
**AJCC stage**																
IIIA	Reference				Reference				Reference				Reference			
IIIB	1.239	0.975	1.576	0.080	1.139	0.700	1.852	0.601	1.324	1.005	1.745	0.046	1.176	0.678	2.039	0.564
**N stage**																
N0	Reference				Reference				Reference				Reference			
N1	0.866	0.535	1.402	0.559	0.928	0.546	1.58	0.784	0.823	0.464	1.460	0.505	0.824	0.427	1.587	0.562
N2	1.232	0.945	1.606	0.122	1.028	0.644	1.642	0.907	1.317	0.973	1.782	0.075	1.047	0.616	1.782	0.865
N3	1.114	0.739	1.678	0.607	-	-	-	-	1.138	0.711	1.821	0.590	-	-	-	-
**Tumor size (cm)**																
<6.0	Reference				Reference				Reference				Reference			
≥6.0	1.530	1.170	2.001	0.002	1.694	1.263	2.273	<0.001	1.714	1.257	2.337	0.001	1.767	1.265	2.470	0.001
**Surgery**																
No	Reference				Reference				Reference				Reference			
Yes	0.464	0.310	0.696	<0.001	0.537	0.337	0.855	0.009	0.423	0.261	0.686	<0.001	0.519	0.291	0.925	0.026
**Radiotherapy**																
No	Reference				Reference				Reference				Reference			
Yes	0.671	0.527	0.854	0.001	0.644	0.472	0.878	0.006	0.721	0.546	0.953	0.022	0.608	0.427	0.866	0.006

We further investigated the prognostic factors in nonsurgical patients (Table 3). Favorable impacts of radiotherapy were observed for nonsurgical patients (OS: HR = 0.6, 95% CI: 0.4–0.8, *P*<0.001; LCSS: HR = 0.6, 95% CI: 0.4–0.8, *P*<0.001; Supplementary Figure S2). Patients older than 66 years were found to have significantly inferior OS (HR = 1.7, 95% CI: 1.2–2.3, *P*=0.001).

**Table 3:** Results of univariate and multivariate Cox regression of prognostic factors for OS and LCSS in nonsurgical T4 SS-NSCLC patients (*n*=337)

Characteristics	OS								LCSS							
	Univariate analysis	95% CI lower	95% CI upper	*P*	Multivariate analysis	95% CI lower	95% CI upper	*P*	Univariate analysis	95% CI lower	95% CI upper	*P*	Multivariate analysis	95% CI lower	95% CI upper	*P*
**Sex**																
Male	Reference				-	-	-	-	Reference				-	-	-	-
Female	0.999	0.779	1.281	0.992	-	-	-	-	0.998	0.752	1.325	0.990	-	-	-	-
**Age (years)**																
<66	Reference				Reference				Reference				-	-	-	-
≥66	1.41	1.103	1.803	0.006	1.683	1.237	2.289	0.001	1.102	0.834	1.455	0.494	-	-	-	-
**Ethnicity**																
Caucasian	Reference				-	-	-	-	Reference				-	-	-	-
African	1.053	0.726	1.527	0.785	-	-	-	-	1.073	0.704	1.634	0.744	-	-	-	-
Other	1.184	0.661	2.12	0.570	-	-	-	-	1.405	0.762	2.591	0.276	-	-	-	-
**Marital status**																
Married	Reference				-	-	-	-	Reference				-	-	-	-
Unmarried	1.138	0.890	1.455	0.303	-	-	-	-	1.103	0.835	1.457	0.489	-	-	-	-
**CHSDA**																
East	Reference				-	-	-	-	Reference				-	-	-	-
Pacific Coast	1.165	0.885	1.533	0.277	-	-	-	-	1.152	0.844	1.571	0.372	-	-	-	-
Northern Plains	1.154	0.795	1.674	0.451	-	-	-	-	1.053	0.681	1.630	0.816	-	-	-	-
Southwest	0.910	0.478	1.731	0.773	-	-	-	-	0.831	0.386	1.787	0.635	-	-	-	-
**Grade**																
Well/Moderate	Reference				-	-	-	-	Reference				-	-	-	-
Poor/Undifferentiated	0.815	0.552	1.204	0.304	-	-	-	-	0.784	0.510	1.205	0.267	-	-	-	-
**Location**																
Left	Reference				-	-	-	-	Reference				-	-	-	-
Right	0.942	0.736	1.205	0.633	-	-	-	-	1.041	0.787	1.377	0.779	-	-	-	-
**Histological type**																
Others	Reference				-	-	-	-	Reference							
SQ	0.887	0.672	1.172	0.400	-	-	-	-	0.857	0.626	1.174	0.337	0.991	0.688	1.428	0.961
AD	0.840	0.610	1.156	0.285	-	-	-	-	0.771	0.534	1.115	0.167	0.838	0.544	1.291	0.423
**AJCC stage**																
IIIA	Reference				-	-	-	-	Reference				-	-	-	-
IIIB	1.114	0.863	1.436	0.407	-	-	-	-	1.177	0.880	1.574	0.273	-	-	-	-
**N stage**																
N0	Reference				-	-	-	-	Reference				-	-	-	-
N1	0.842	0.499	1.420	0.518	-	-	-	-	0.713	0.376	1.354	0.301	-	-	-	-
N2	1.110	0.837	1.470	0.469	-	-	-	-	1.152	0.838	1.585	0.383	-	-	-	-
N3	0.971	0.639	1.477	0.892	-	-	-	-	0.964	0.597	1.554	0.879	-	-	-	-
**Tumor size (cm)**																
<6.0	Reference				Reference				Reference							
≥6.0	1.334	1.003	1.775	0.048	1.617	1.202	2.175	0.002	1.474	1.061	2.047	0.021	1.602	1.148	2.236	0.006
**Radiotherapy**																
No	Reference				Reference				Reference							
Yes	0.578	0.448	0.746	<0.001	0.576	0.422	0.787	0.001	0.616	0.459	0.825	0.001	0.525	0.370	0.745	<0.001

## Discussion

The last decade has witnessed an evolution in the management and surgical approach for SS-NSCLC, but survival at 5 years from T4 tumors remains approximately 30% [3,8,19,20]. In addition, more than half of patients with T4 SS-NSCLC are not eligible for trimodality treatment [7–9,21]. Therefore, in this subgroup of SS-NSCLC patients, we not only focussed on operable candidates, but also paid more attention to patients who were not candidates for resection.

In this population-based study, approximately 70% of the T4 SS-NSCLC patients received radiotherapy, therapy involving surgery was utilized in only a small proportion of patients, which decreased (20 to 2%) as the N status increased. Nonetheless, our study still demonstrated the remarkable survival benefit when patients can be selected for surgery, especially in the N0-1 subgroup. In accordance with previously published reports [5,22], lobectomy was associated with superior survival, while no additional benefits on long-term survival were provided by radiotherapy irrespective of the treatment sequence.

In the current study, the median OS was 12 months with a 5-year OS rate of 15.8%, which was obviously inferior to the SWOG 9806/JCOG 9416 trial [3,4] and recently published retrospective analysis [8] in T4 SS-NSCLC, which may be attributed to the high proportion of N2-3 patients (56.6%). However, our survival results from the group undergoing surgery were not less than those of the above studies. It is still controversial whether N2 disease is a contraindication for surgical resection for SS-NSCLC [22,23]. Herein, the OS and LCSS of the treatment group which underwent surgery appears to be numerically superior to radiotherapy alone, but the difference was not significant. These results are difficult to interpret because of the retrospective nature of the study, the small number of surgical patients, and other confounding factors. Moreover, the protective role of radiotherapy was also validated for nonsurgical T4 SS-NSCLC patients (Tables 2 and 3; Supplementary Figure S2), which has not been noted in other studies. Until now, given the low resectability in T4N2 SS-NSCLC patients, radiotherapy has been recommended [24,25].

Defining the prognostic factors for survival in surgical and nonsurgical patients with SS-NSCLC is a challenging topic [2,8,26–28]. Tumor size, though not mentioned by previous studies on SS-NSCLC, has been proven as an independent risk factor of decreased OS and LCSS for T4 SS-NSCLC patients. In fact, larger tumor size, indicating a higher likelihood of involving adjacent critical structures, can limit the possibility of complete resection [29]. Furthermore, because the tumor load cannot be substantially alleviated under conservative therapy, it seems reasonable that larger tumor size is associated with worse prognosis for nonsurgical patients. Because T4N0 SS-NSCLC has been reclassified into stage IIIA disease [30], the findings of Morgensztern et al*.*, who investigated the prognostic significance of tumor size in patients with stage III NSCLC, further confirms our results using a larger sample [31]. In addition to tumor size, increasing age was also associated with worse OS in the entire group, which may be the result of the weaker physical condition and shorter residual lifespan of elderly patients. Another possible explanation is that older patients are more likely to have age-related comorbid conditions and risk increases with age [32–34]. In addition, OS was independently affected by marital status in the entire group, which was consistent with several previous studies on the prognostic factors for lung cancer patients [31,32,35].

Our study is inevitably limited by its retrospective nature. Because information regarding the detailed chemotherapy and radiotherapy regimens was NA in the SEER database, not only are we unable to comment on the role of either induction chemotherapy or adjuvant chemotherapy, but we also have been unable to study the relationships between prognoses and different radiotherapy regimens. Second, although we had a relatively small number of surgical patients, our study was still amongst the larger series investigating T4 SS-NSCLC. Therefore, further investigation of the prognostic factors for surgical patients was hampered when using the Cox regression model. Meanwhile, because PSM was performed, the subsequently small number of nonsurgical patients could have led to nonsignificance of other prognostic factors. Moreover, the staging of nonsurgical patients was based on the clinical stage rather than the pathological stage, which was another important confounding factor in our study. Finally, progression-free survival (PFS) may be an important prognosis marker for this cohort, but we could not obtain information concerning PFS in the SEER database.

In conclusion, our study showed that T4 SS-NSCLC patients had a poor prognosis and that treatment remains a challenge. Surgical resection remains the best option for those with resectable disease. For nonsurgical T4 SS-NSCLC patients, radiotherapy should be actively considered. New treatment strategies and the addition of targets and/or immune agents is warranted.

## Supporting information

**Supplementary Figure 1:** Overall (A) and lung cancer-specific survival (B) with regard to radiotherapy in patients who underwent surgery.

**Supplementary Figure 2:** Overall (A) and lung cancer-specific survival (B) with regard to radiotherapy in patients without surgery.

**Supplementary Table 1.** Basic characteristics for T4 SS-NSCLC patients with and without surgery before and after a propensity-matched analysis

**Supplementary Table 2.** Landmark analyses showing the association of survival with radiotherapy in patients who underwent surgery and who survived a minimum of ≥ 12 or ≥ 24 months.

## Abbreviations

AJCC: American Joint Committee on Cancer
CI: confidence interval
CRT: chemoradiotherapy
ICD-O-3: International Classification of Diseases for Oncology 3rd Edition
JCOG: Japan Clinical Oncology Group
LCSS: lung cancer-specific survival
NA: not available
NRT: neoadjuvant radiotherapy
OS: overall survival
PFS: progression-free survival
PORT: postoperative radiotherapy
PSM: propensity score matching
SEER: Surveillance, Epidemiology, and End Results database
SS-NSCLC: superior sulcus non-small cell lung cancer
SWOG: Southwest Oncology Group
