# Supplementary material for: Treatment of clinical T4 stage superior sulcus non-small cell lung cancer: a propensity-matched analysis of the surveillance, epidemiology, and end results database
Source: Biosci Rep. 2019 Feb 1;39(2):BSR20181545. doi: 10.1042/BSR20181545 (PMC6356038; doi:10.1042/BSR20181545)
Supplement: Supplementary file 1 [file bsr20181545_Supp1.pdf]

Supplementary Figure 1. Overall (A) and lung cancer-specific survival (B) with regard to radiotherapy in patients who underwent surgery.

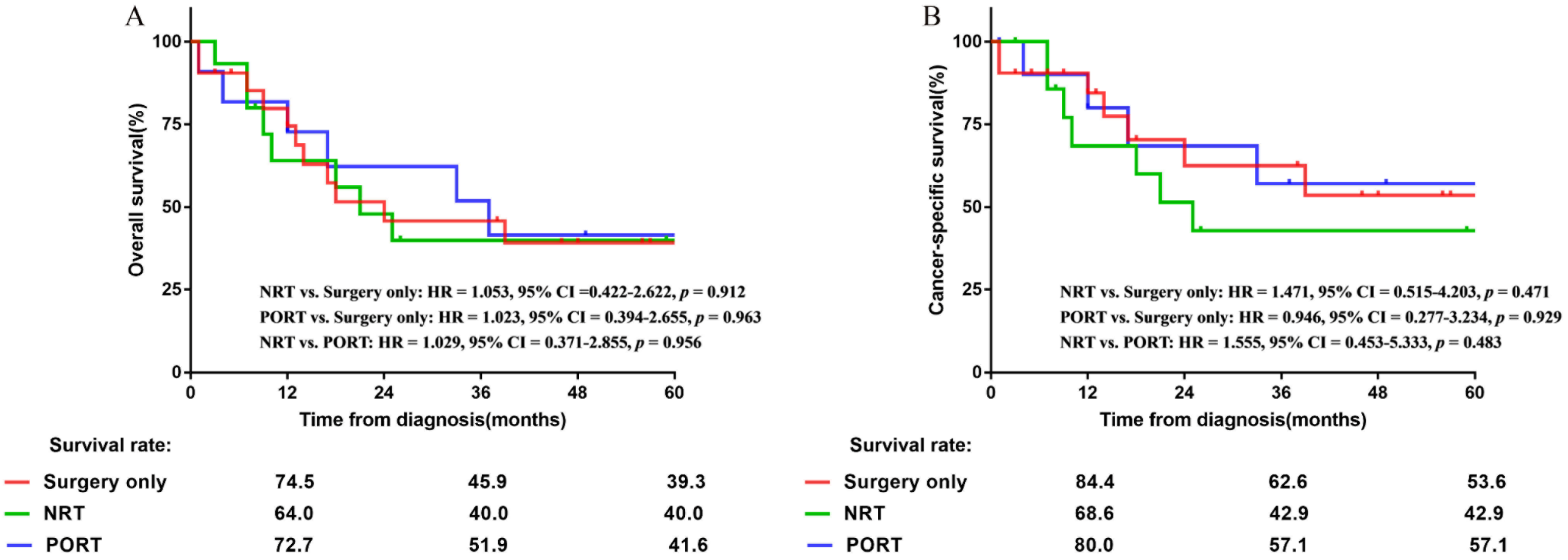

Supplementary Figure 2. Overall (A) and lung cancer-specific survival (B) with regard to radiotherapy in patients without surgery.

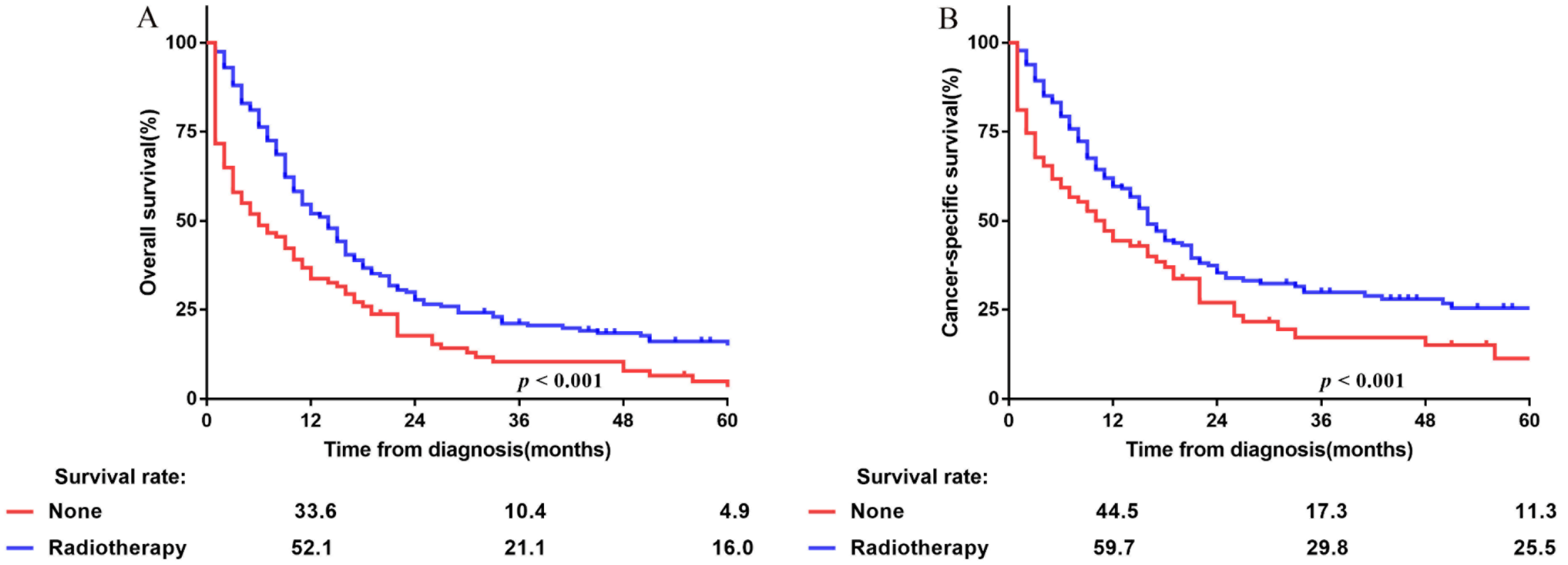

Supplementary Table 1. Basic characteristics for T4 SS-NSCLC patients with and without surgery before and after a propensity-matched analysis

	Entire population					Propensity-matched popuplation				
**Characteristics**	**Surgery (+)** (n=47)	**%**	**Surgery (-)** (n=337)	**%**	***p***	**Surgery (+)** (n=47)	**%**	**Surgery (-)** (n=47)	**%**	***p***
**Gender**					0.546					0.289

Male	26	55.3	202	59.9		26	55.3	32	68.1	
Female	21	44.7	135	40.1		21	44.7	15	31.9	
**Age (years)**					0.819					0.845
Mean (SD)	65.96	±11.34	66.4	±11.77		65.96	±11.34	66.45	±11.84	
**Ethnicity**					0.043					0.677
Caucasian	45	95.7	280	83.1		45	95.7	43	91.5	
African	0	0	39	11.6		0	0	0	0	
Other	2	4.3	18	5.3		2	4.3	4	8.5	
**Marital status**					0.059					0.144
Married	28	63.6	159	48.5		28	63.6	22	47.8	
Unmarried	16	36.4	169	51.5		16	36.4	24	52.2	
Unknown	3		9			3		1		
**CHSDA**					0.429					0.053
East	26	55.3	167	49.6		26	55.3	19	40.4	
Pacific Coast	17	36.2	115	34.1		17	36.2	16	34	
Northern Plains	2	4.3	43	12.5		2	4.3	11	23.4	
Southwest	2	4.3	13	3.9		2	4.3	1	2.1	
**Grade**					0.157					0.800
Well/Moderate	16	40	47	28.5		16	40	11	44	
Poor/Undifferentiated	24	60	118	71.5		24	60	14	56	
Unknown	7		172			7		22		
**Location**					0.585					0.837
Left	23	48.9	178	52.8		23	48.9	24	51.1	
Right	24	51.1	154	45.7		24	51.1	22	46.8	
Bilateral	0	0	5	1.5		0	0	1	2.1	

**Histological type**					0.048					1.000
SQ	20	42.6	116	34.4		20	42.6	21	44.7	
AD	16	34	80	23.7		16	34	16	34	
Others	11	23.4	141	41.8		11	23.4	10	21.3	
**AJCC stage**					0.001					1.000
IIIA	31	66	129	40.1		31	66	31	66	
IIIB	16	34	193	59.9		16	34	16	34	
Unstaged	0		15			0		0		
**N stage**					0.005					0.795
N0	26	55.3	104	32.3		26	55.3	26	55.3	
N1	5	10.6	25	7.8		5	10.6	5	10.6	
N2	15	31.9	152	47.2		15	31.9	13	27.7	
N3	1	2.1	41	12.7		1	2.1	3	6.4	
Nx	0		15			0		0		
**Tumor size (cm)**					<0.001					0.102
Mean (SD)	4.6	±2.2	6.4	±2.8		4.6	±2.2	5.5	±2.1	
**Radiotherapy**					0.071					1.000
Yes	26	55.3	231	68.5		26	55.3	26	55.3	
No	21	44.7	106	31.5		21	44.7	21	44.7	

Supplementary Table 2. Landmark analyses showing the association of survival with radiotherapy in patients who underwent surgery and who survived a minimum of ≥ 12 or ≥ 24 months.

Factors	OS				LCSS			
	≥ 12-month survivors		≥ 24-month survivors		≥ 12-month survivors		≥ 24-month survivors	
	HR(95%CI)	p value	HR(95%CI)	p value	HR(95%CI)	p value	HR(95%CI)	p value

Surgery only	Reference		Reference		Reference		Reference	
NRT	0.645(0.166-2.502)	0.526	0.794(0.072-8.807)	0.851	0.916(0.218-3.846)	0.905	0.809(0.073-8.962)	0.863
PORT	1.049(0.329-3.342)	0.935	1.905(0.310-11.709)	0.487	0.937(0.224-3.926)	0.929	0.733(0.066-8.105)	0.800
